# Is there still hesitancy towards SARS-CoV-2 vaccination among people with neurological disease– a survey of the NeuroCOVID-19 task force of the European Academy of Neurology

**DOI:** 10.1007/s10072-025-08017-w

**Published:** 2025-02-04

**Authors:** Sonja Hochmeister, Martin Rakusa, Elena Moro, Daniel Bereczki, Francesco Cavallieri, Alessandra Fanciulli, Saša R. Filipović, Alla Guekht, Raimund Helbok, Filippo Martinelli Boneschi, Serefnur Özturk, Alberto Priori, Barbara Willekens, Dauren Ramankulov, Johann Sellner

**Affiliations:** 1 Department of Neurology, Medical University of Graz, Graz, Austria; 2 Division of Neurology, University Medical Centre Maribor, Maribor, Slovenia; 3 Division of Neurology, Grenoble Alpes University, CHU of Grenoble, Grenoble Institute of Neurosciences, INSERM U1216, Grenoble, France; 4 Department of Neurology, Semmelweis University, Budapest, Hungary; 5 Neurology Unit, Neuromotor & Rehabilitation Department, Azienda USL-IRCCS di Reggio Emilia, Reggio Emilia, Italy; 6 Department of Neurology, Medical University of Innsbruck, Innsbruck, Austria; 7 Institute for Medical Research, University of Belgrade, Belgrade, Serbia; 8 Research and Clinical Center for Neuropsychiatry, Moscow, Russian Federation; 9 Pirogov Russian National Research Medical University, Moscow, Russian Federation; 10 Department of Neurology, Kepler University Hospital, Johannes Kepler University, Linz, Austria; 11 Neurology Unit, ASST Santi Paolo e Carlo, Milan, Italy; 12 Department of Health Sciences, University of Milan, Milan, Italy; 13 Department of Neurology, Faculty of Medicine, Selcuk University, Konya, Turkey; 14 ’’Aldo Ravelli’’ Center for Neurotechnology and Experimental Brain Therapeutics, Department of Health Sciences, University of Milan, Milan, Italy; 15 Clinical Neurology Unit, Department of Health Sciences, “Azienda Socio-Sanitaria Territoriale Santi Paolo E Carlo”, University of Milan, Milan, Italy; 16 Department of Neurology, Antwerp University Hospital, Edegem, Belgium; 17 Translational Neurosciences Research Group, University of Antwerp, Wilrijk, Belgium; 18 European Academy of Neurology, Vienna, Austria; 19 Department of Neurology, Landesklinikum Mistelbach-Gänserndorf, Liechtensteinstrasse 67, Mistelbach, 2130 Austria; 20 Karl Landsteiner University of Health Sciences, Krems, Austria; 21 Department of Neurology, Christian Doppler Medical Center, Paracelsus Medical University, Salzburg, Austria; 22 Department of Neurology, Klinikum rechts der Isar, School of Medicine, Technische Universität München, München, Germany

**Keywords:** COVID-19, SARS-CoV-2, Vaccination hesitancy, Disease prevention, Neurological disease, Public health, Multiple sclerosis, Autoimmune

## Abstract

**Background:**

An online 3-item survey was sent to the European Academy of Neurology (EAN) community and inquired about the persistence of SARS-CoV-2 vaccination skepticism and the underlying thoughts and factors restricting vaccine use among patients with neurological conditions.

**Results:**

We obtained 616 responses from 84 countries, predominantly from Europe. In the view of the treating neurologist, patients with multiple sclerosis (MS), neuroimmunological disorders (ND), and chronic neurological infections continued to have high levels of skepticism toward SARS-CoV-2 vaccination. Patients with MS/ND were quoted as the most hesitant group, with 60% of the respondents sharing this impression. The patient group perceived as most confident towards immunization against COVID-19 and with the lowest level of distrust towards the vaccine were those with sleep disorders. For all other conditions, perceived distrust ranged between 42 and 52%. Fear of adverse events of vaccination or disease reactivation was perceived by 87% of patients with MS/ND and more than 70% of patients with stroke/vascular neurology, neuromuscular disorders, chronic neurological infections, and peripheral neuropathy. Patients with sleep disorders (54%), autonomic disorders (46%), movement disorders (43%), and dementia (43%) were sensed as less fearful of vaccine-related adverse events.

**Conclusion:**

Despite the large body of evidence proving the efficacy and safety of SARS-CoV-2 vaccination, patients with certain neurological disorders still have a surprisingly high percentage of distrust and fear of adverse events. Our observations emphasize the importance of continuous evidence-based information delivery and patient education by treating neurologists.

**Supplementary Information:**

The online version contains supplementary material available at 10.1007/s10072-025-08017-w.

## Key summary points

• In 2022, we observed increased rates of vaccination hesitancy and skepticism for SARS-CoV-2 vaccination among patients with certain neurological disorders.
• We aimed to reassess the considerations by contacting neurology residents and specialists.
• In 2023, patients with MS and autoimmune neurological disorders continue to show distrust and fear of adverse events.
• Physicians play a crucial role in supporting these public health efforts, which have come further under scrutiny during the pandemic.

**Supplementary Information:**

The online version contains supplementary material available at 10.1007/s10072-025-08017-w.

## Introduction

The COVID-19 pandemic claimed 7 million deaths among 675 million cases from its start in late 2019 and the official end in early May 2023, as declared by the World Health Organization (WHO) [1]. During this time, more than 13.3 billion vaccine doses were administered. With the need to treat the overflowing COVID-19 cases in the early phase of the pandemic, clinical services had to be cut back in almost all medical subspecialties, including the out- and inpatient care of patients with neurological disorders [2, 3]. Of note, neurological complications of COVID-19 led to a rise in patients with acute consultations and the need for hospital admission [4–6]. Moreover, studies corroborated that a subgroup of patients with neurological diseases are at high risk for severe and complicated COVID-19 disease course [7–10]. This observation early in the pandemic was alarming concerning rapid diagnosis and appropriate treatment for neurological conditions. However, it was surprising that a recent European Academy of Neurology (EAN) survey study performed in early 2023 provided evidence for continued restrictions in daily clinical neurological practice [11].

Our pilot study in 2022 disclosed an unexpectedly high rate of hesitancy and skepticism for SARS-CoV-2 vaccination among patients with certain neurological disorders despite evidence from randomized controlled trials on efficacy for preventing COVID-19 and severe and fatal courses [12]. Controversy on immunization, however, is not new, and vaccines have been questioned again with the approval of the COVID-19 vaccines. Vaccine hesitancy is complex and multi-faceted, and vaccine confidence is volatile and time-specific [13]. Indeed, the determinants of vaccine hesitancy include historical period, geographical area, political situation, complacency, convenience, and confidence in vaccines. Notably, vaccine hesitancy prospectively predicts nocebo side effects following COVID-19 vaccination [14]. The most frequent reasons for vaccination hesitancy in our study were the fear of worsening the pre-existing neurological illness, drug interaction, and the risk of vaccination-related adverse events. This finding is remarkable, as national and governmental bodies and medical societies have continuously provided statements about the favorable risk-benefit profile of SARS-CoV-2 vaccination, particularly in neurology [15, 16]. Interestingly, multiple sclerosis (MS) and other neuroimmunological disorders (ND) were the front runners for vaccination hesitancy and skepticism in this and other surveys [17–19]. Individuals with motor neuron disease, spinal cord injury, traumatic brain injury, and neuro-oncological diagnoses were, on the other hand, less likely to face vaccination with skepticism.

Here, we aimed to reassess the considerations of patients with neurological diseases towards the SARS-COV2 vaccination and potential reasons for continued skepticism in 2023 by contacting neurology residents and specialists within the EAN community.

## Methods

### The survey preparation

The EAN NeuroCOVID-19 task force for a survey study on “Inequities for prevention, diagnosis, and treatment of COVID-19” was approved by the Scientific Committee of the EAN. The survey was divided into two parts, with the first focusing on managing the healthcare crisis related to COVID-19 and the second examining vaccine hesitancy among patients with neurological conditions. This study deals with the results of the second part of the survey, and the survey questions are available in the supplemental material.

Three task force members prepared the first draft, which was presented to other members and revised five times before final approval by the Task Force. The survey was prepared with SurveyMonkey (San Mateo, CA, USA) and available online from April 1st to May 1st 2023. We approached the EAN community by placing information about the survey in the monthly EAN newsletter. The newsletter is distributed to EAN members and patients who registered. Participants could opt for a lottery for free registration at the 9th EAN Congress in Budapest.

### Data analysis

For the analysis, we used descriptive statistics; figures and charts were assembled in Microsoft Excel 2021 (Redmond, CA, USA). Participants could choose not applicable (N/A) in some questions. Therefore, we excluded those answers and adjusted the final results. A table with the number of the N/A for each question is available in the supplemental file. To evaluate the reliability and variability of the survey results, we calculated the Weighted Average Score (WAS), standard deviation, standard error (SE), and 95% confidence intervals (CI). Answers were weighted from 1 (of little importance) to 4 (absolutely important), or five points from strongly disagree to strongly agree.

### Ethics declaration

No ethics approval was required for this anonymized online survey study according to Austrian regulations.

## Results

Of 1231 recipients of the newsletter, 616 (50%) completed the survey.

### Demographic data

**Fig. 1:**
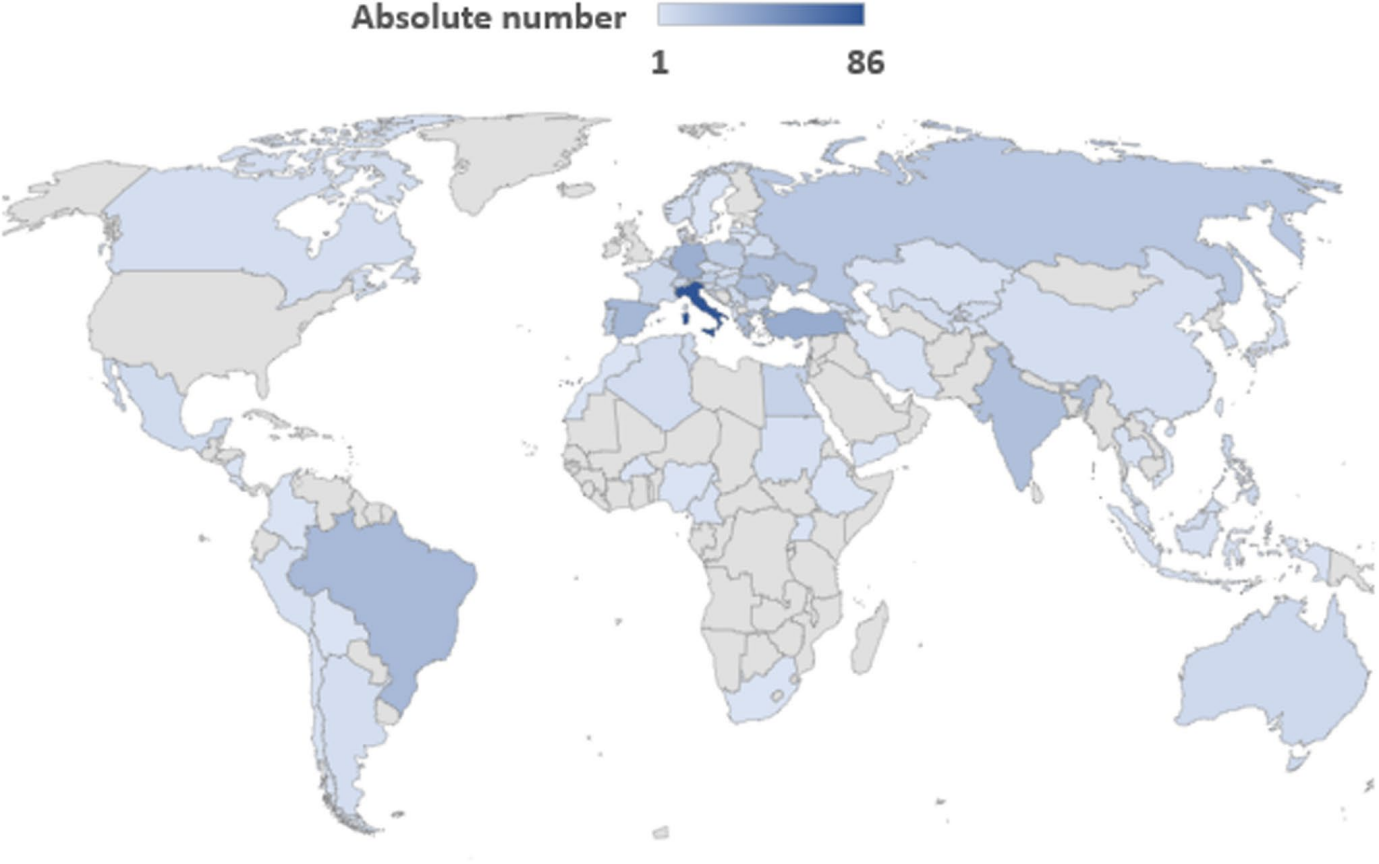
Geographical distribution of participants

The survey reached 84 countries worldwide (Fig. 1); demographic data of the respondents to the survey is given in Table 1; a detailed list of repondents’ countries of practice is given in Suppl. Table 1). From a continental viewpoint, the majority of the participants were from Europe, followed by Asia, South America, and Africa. Italy had the highest representation among the countries, with 14% of respondents. Other European countries with relatively higher representation were Turkey (6%), Germany (5%), Greece, Romania, Spain and Ukraine (all 4%). Non-European respondents mainly came from India (4%). Several countries had only one respondent, e.g., Bolivia, Burkina Faso, Colombia, Costa Rica, Ethiopia, Kyrgyzstan, Lebanon, Montenegro, Morocco, Nicaragua, Nigeria, South Africa, Sweden, Taiwan, Tajikistan, Uganda, Vietnam, and Yemen.

**Table 1:** Demographic data of the 616 respondents of the survey

	*N*	Percentage
Female	338	55%
**Age range (years)**		
18–24	17	3%
25–34	259	42%
35–44	146	24%
45–54	104	17%
55–64	57	9%
65 and older	33	5%
**Years in practice**		
Neurology resident	210	34%
Neurologist of 5 years	152	25%
Neurologist of 10 years	64	10%
Neurologist of 15 years	31	5%
Neurologist of more than 15 years	159	26%
**Place of work** ^**a**^		
University hospital	347	41%
Public hospital	201	24%
Private hospital	74	9%
Research facility	58	7%
Private practice	74	9%
Outpatient clinic	58	7%
Other	26	3%
**Most frequent neurological fields of interest** ^**b**^		
Stroke/vascular neurology	243	16%
Peripheral Nephropathy	74	5%
Neuromuscular disorders	104	7%
Neurological emergency	68	4%
Movement disorders	155	10%
Multiple sclerosis/neuroimmunology	183	12%
Dementia/cognitive disorders	128	8%
Epilepsy	134	9%
Headache and pain	128	8%
Neuroinfection	34	2%
Sleep disorders	49	3%
Autonomic nervous system	17	1%
Neurorehabilitation	48	3%
Neurocritical care	29	2%
General neurology	120	8%
Other	33	2%

^a^multiple choices possible; ^b^maximum three choices

Males and females were equally represented (Table 1). Two-thirds of participants were younger than 44 years. Most participants were between 25 and 34 years old (42%). The second largest age group was between 35 and 44 years of age (24%) (Table 1).

### Fields of expertise

Most respondents were residents (34%), followed by neurologists with more than 15 years of experience (26%). 72% of participants worked partially in a university, public or private hospital. The most represented fields of expertise were stroke/vascular neurology (16%), multiple sclerosis/neuroimmunology (12%) and movement disorders (10%). The least frequent fields of interest among the participants were autonomic nervous system disorders (1%), neurocritical care and neuroinfection (both 2%), and neurorehabilitation (3%) (Table 1).

### Measures of the reliability and variability of the survey results

Variances, SD and SE were small to moderate for most neurological conditions, and CIs were narrow (supplement tables S2). Regarding hesitancy to receive SARS-CoV-2 vaccination, the average WAS significantly higher for multiple sclerosis and neuroimmunology and the lowest for sleep disorders.

When asked for the reasons for vaccine hesitancy (distrust in SARS-CoV-2 vaccination, fear of adverse vaccination events, and fear of disease reactivation or worsening), the average WAS was significantly higher for multiple sclerosis and neuroimmunology than for the rest of the neurological conditions and the lowest for sleep disorders. Heat maps of the WAS are presented in Fig. 4.

### Results of the survey

**Fig. 2:**
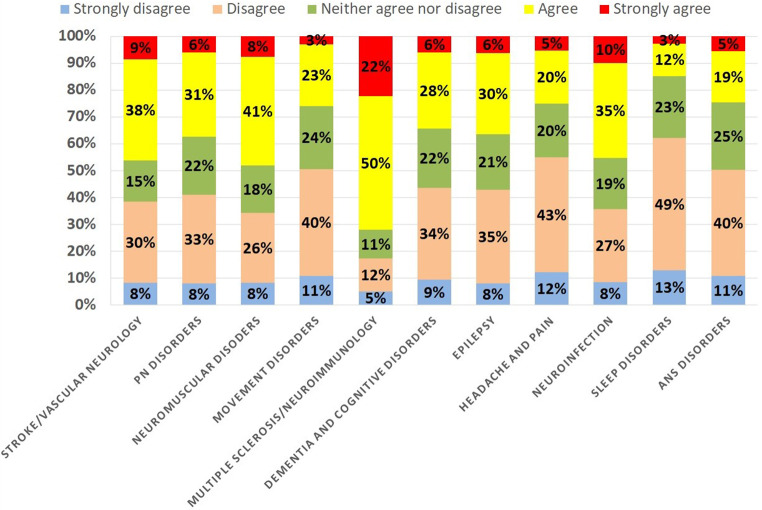
Hesitancy to receive SARS-CoV-2 vaccination among people with neurological conditions compared to healthy people of the same age

There was heterogeneity for vaccination hesitancy among different neurological conditions. The most common condition associated with a reluctance to SARS-CoV-2 vaccination was multiple sclerosis/neuroimmunology, followed by patients with infectious diseases of the CNS and neuromuscular disorders (Fig. 2). From all neurological conditions, patients with multiple sclerosis/neuroimmunological disorders were the most hesitant to receive SARS-CoV-2 vaccination in comparison to healthy people of the same age- as much as 72% of the participants agreed or strongly agreed. The hesitancy was the lowest among patients with sleep disorders, where only 3% of participants strongly agreed, and 12% agreed.

**Fig. 3:**
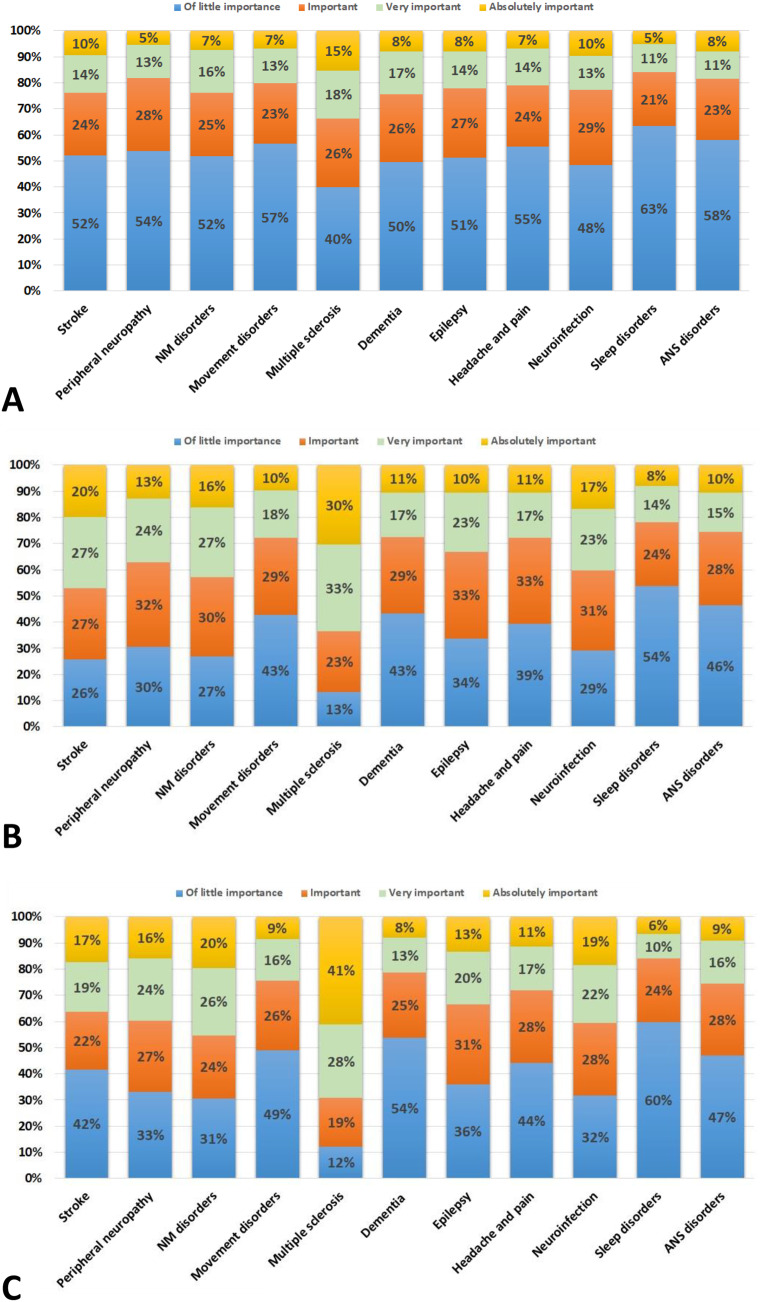
The reasons for vaccine hesitancy among people with neurological conditions from top to bottom: distrust in SARS-CoV-2 vaccination (**A**), fear of adverse events of vaccination (**B**), fear of disease reactivation/worsening (**C**). Stroke– stroke/vascular neurology; NM– neuromuscular; Multiple sclerosis– multiple sclerosis/neuroimmunology; ANS - Autonomic nervous system. %– proportion of answers

The next part of the survey aimed to elucidate the reasons for this hesitancy (Fig. 3). Patients with multiple sclerosis/neuroimmunological disorders had the highest rate of distrust in the efficacy and safety of the SARS-CoV-2 vaccination (59% pooled replies of “absolutely important”, “very important” and “important”). For all other conditions, distrust ranged between 42% and 52%, again with the lowest rate observed for sleep disorders (37%) (Fig. 3A).

Similar findings were observed when asking about fear of adverse events caused by vaccination (Fig. 3B). 86% of respondents agreed that patients with multiple sclerosis/ neuroimmunological disorders were afraid of vaccine adverse events (Fig. 3B).

For four other neurological conditions (stroke/vascular neurology, neuromuscular disorders, neuroinfection, and peripheral neuropathy), at least 70% of respondents replied that their patients were afraid of vaccine-related adverse events. On the other hand, patients with sleep disorders (54%), autonomic disorders (46%), movement disorders (43%), and dementia (43%) considered vaccination-related adverse events of minor importance and had the least fear (Fig. 1B).

**Fig. 4:**
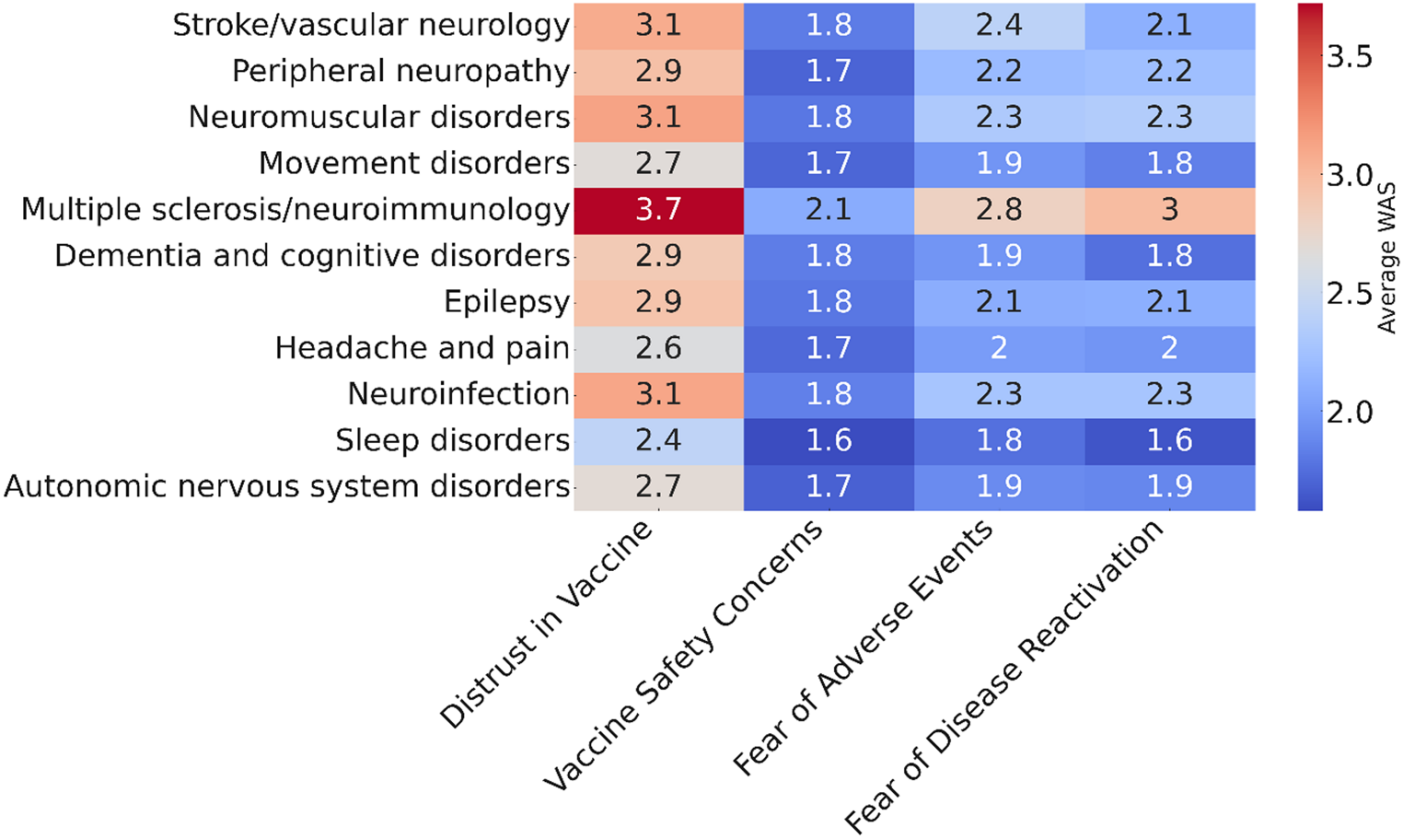
Heatmaps of average Weighted Average Score (WAS) for neurological conditions

Similar observations were made when inquiring about fear of reactivation or worsening of the underlying neurological disease (Fig. 3C). Patients with multiple sclerosis/neuroimmunological disorders were most worried (pooled agreement of 88%), whereas only 12% ascribed little importance to this issue. The fear was three times more common in patients with Multiple sclerosis than in patients with epilepsy (36%) and even five times stronger than in patients with sleep disorders (60%) (Fig. 3C) (Fig. 4).

## Discussion

Vaccination hesitancy and skepticism acuminated with the roll-out of the global COVID-19 immunization efforts, the most extensive public health campaign in history (8–10). This observation comes unexpectedly, as the various vaccine preparations have unequivocally shown to be the most efficient measure in phase 3 trials to prevent COVID-19 infection and hospitalization and mortality related to the disease [20, 21]. Notably, patients with neurological comorbidities were mainly excluded from the pivotal COVID-19 vaccination trials; therefore, at the time the results of the trials became available, many of the concerns raised by this group of patients could not be refuted on the basis of the study evidence. However, the efficacy and safety of vaccination against SARS-CoV-2 was proven in subsequent real-world studies for the elderly population and patients with chronic diseases and weakened immune system [22–24]. Furthermore, reports of the infrequent occurrence of cerebral venous sinus thrombosis in association with a vector-based type of COVID-19 vaccine might have particularly intensified the fear of further brain damage in persons with pre-existing nervous system disorders. Moreover, the temporary suspension of the distribution of vector-based vaccines and the lack of clinical experience with mRNA vaccines may have decreased public vaccine acceptance in general and especially in our patients [25, 26].

In a previous study from 2022, we found a surprisingly high hesitancy of getting vaccinated against Covid-19 among patients with neurological diseases, especially those with multiple sclerosis and neuroimmunological disorders. In the current study, we aimed to revisit the pilot trial observations in a subsided pandemic situation in spring 2023. The survey was conducted at a time of infrequent severe COVID-19 disease courses, mostly related to the circulation of less virulent SARS-CoV-2 variants and the establishment of immunity in the general population due to vaccination and previous encounters with SARS-CoV-2 variants. However, our data from replies of over 600 neurology specialists and neurologists in training worldwide showed a similar picture of vaccination hesitancy and skepticism as identified in the pilot study. Again, the highest rates of hesitancy and skepticism were present among patients with MS or neuroimmunological diseases [12]. This observation needs to be questioned critically after millions of vaccination doses were administered and were critical to end the pandemic. Our findings are in line with an Australian online survey of patients with MS, which disclosed general and MS-specific concerns about COVID-19 vaccination [20]. Yet, they also observed high vaccination rates, implicating that the concerns did not translate to clinical practice. Interestingly, greater MS-specific concerns were reported by those who had suboptimal disease control and impaired daily activities. The now scientifically disproved association between vaccination and MS is likely to be blamed for the prevailing vaccination hesitancy in patients with MS [21]. A study of German ambulatory claims data from 2005 to 2018 revealed that patients with MS were less likely to be vaccinated compared to controls and patients with other autoimmune disorders five years after diagnosis [22]. There are even data that implicate that vaccination is associated with a lower likelihood of being diagnosed with MS within the next five years [21]. Our findings contrast a study in rheumatic disease, where the vaccination rate increased to 95% in 2022 from 83.4% in 2021 [23]. The rheumatic disease data were retrieved from two large international, multicenter patient self-reported online surveys, whereas we summarized the feedback from the treating physicians. Interestingly, people with idiopathic inflammatory myopathies in the rheumatologic disease cohort reported higher levels of skepticism, primarily due to safety concerns. This observation is paradoxical since these patients frequently have interstitial lung diseases and are at risk for respiratory infection. There is an analogy with the higher risk for unfavorable COVID-19 outcomes of patients with MS who have higher levels of neurological disability [24]. Of note, in patients with MS, there is also no increase in the relapse rate following SAS-CoV-2 vaccination [25, 26]. Most importantly, the long-awaited confirmation that SARS-CoV-2 vaccination protects from severe and fatal COVID-19 courses among patients with MS needs to be emphasized more openly [27]. In analogy, well-conducted epidemiological studies could not confirm the causal relationship for autoimmune disorders other than MS, and there is no evidence for a worsening of the pre-existing disease by SARS-CoV-2 vaccination [28, 29].

Physicians are central in building trust in vaccination and counseling for concerns. However, although the pandemic has markedly subsided, the clinical service has still not fully recovered to the pre-pandemic situation, as our recent study performed in early 2023 revealed [11]. It could be argued that the persistent lack of personal contact with the treating physician reduced the maintainance of a solid and trusting working relationship with the patients and patient education by physicians and healthcare workers. This knowledge may have been even filled with unsubstantiated opinions and misinformation from social media sources and vaccine-skeptic physicians [30, 31]. Reestablishing patient-physician and patient-nursing-staff interaction at the pre-pandemic level appears essential. The current shortage of medical personnel will undoubtedly pose a further challenge in this respect. The emerging role of telemedicine for keeping patients and healthcare professionals in contact, especially those living in geographically underserved areas or with physical barriers due to disability, may pose a silver lining of the pandemic to be further developed in the future.

Immunization is an integral part of public health, and the concerted action plans protect from deadly disease outbreaks and continue to save millions of lives [15, 32]. Long-term benefits of vaccination include improved educational and economic stability resulting from reduced school and work absenteeism, fewer healthcare visits, and decreased hospitalizations due to preventable illnesses. The use of vaccines, however, decreased in the wake of the pandemic. The consequences are alarming. From November to December 2023, US hospitalization rates increased by 200% for influenza, 51% for COVID-19, and 60% for respiratory syncytial virus (RSV) among all age groups, according to a Centers for Disease Control and Prevention (CDC) advisory [33]. Moreover, there are upsurges in measles cases due to the disruption of routine immunizations worldwide, with a 30-fold increase in Europe [34, 35].

The main limitation of the current study is that we asked the treating neurologists about their interaction and experience with the respective patient’s group and not the respective patients’ group themselves. It cannot, therefore, be compared to other surveys which poll patients directly. Other limitations include use of the respective survey questions as part of a larger survey, which may affect bias; we therefore included questions and data of the full survey to the supplementary data. From a statistical point of view however, SE values were generally low, and 95% CIs were narrow. Therefore, we assume that our results are precise and support our interpretations.

A further limitation is the relatively small number of responses from experts in the fields of autonomic nervous system disorders, neurocritical care, neuroinfectious diseases, and neurorehabilitation, as well as the major focus on Europe. Given the consistency in the replies from the pilot study and the current and other patient-centered surveys on this topic, we however are confident to cover at least the most crucial patient motives.

## Conclusions

From our study we conclude that patients with pre-existing neurological disorders, particularly those with MS or autoimmune neurological disorders, have an increased demand for information about the safety of vaccines, not only for the specific Covid-19 vaccine, but in fact with respect to all vaccinations. The physician plays a crucial role in supporting these public health efforts, which have come further under scrutiny during the pandemic. In the case of COVID-19, the treating physicians need to be aware of the interplay between perceived disease vulnerability, fear of COVID-19, worsening of the underlying neurological disease, and subsequent vaccine hesitancy. The establishment of a solid and trusting physician-patient relationship cannot be overestimated, not only on individual, but also on level of public health care.

## Electronic supplementary material

Below is the link to the electronic supplementary material.

Supplementary Material 1: Supplemental Table 1. Countries of origin of survey’s participants. Supplemental Table 2: The Weighted Average Score (WAS), standard deviation, standard error (SE), and 95% confidence intervals (CI) of SE for vaccine hesitancy.

Supplementary Material 2 Questionaire

## Data Availability

All data generated or analyzed during this study are included in this published article.
