# Supplementary material for: Is there still hesitancy towards SARS-CoV-2 vaccination among people with neurological disease– a survey of the NeuroCOVID-19 task force of the European Academy of Neurology
Source: Neurol Sci. 2025 Feb 4;46(4):1467–76. doi: 10.1007/s10072-025-08017-w (PMC11920348; doi:10.1007/s10072-025-08017-w)
Supplement: Supplementary file 1 — Supplementary Material 1: Supplemental Table 1. Countries of origin of survey’s participants. Supplemental Table 2: The Weighted Average Score (WAS), standard deviation, standard error (SE), and 95% confidence intervals (CI) of SE for vaccine hesitancy. [file 10072_2025_8017_MOESM1_ESM.docx]

**Supplement table 1. Countries of origin of survey's participants**

| Albania | 0,32% | 2 |
| --- | --- | --- |
| Algeria | 0,81% | 5 |
| Argentina | 0,65% | 4 |
| Armenia | 1,14% | 7 |
| Australia | 1,14% | 7 |
| Austria | 2,27% | 14 |
| Azerbaijan | 0,32% | 2 |
| Belarus | 0,97% | 6 |
| Belgium | 2,27% | 14 |
| Bolivia | 0,16% | 1 |
| Brazil | 4,22% | 26 |
| Bulgaria | 0,65% | 4 |
| Burkina Faso | 0,16% | 1 |
| Cameroon | 0,32% | 2 |
| Canada | 0,65% | 4 |
| Chile | 0,32% | 2 |
| China | 0,65% | 4 |
| Colombia | 0,16% | 1 |
| Costa Rica | 0,16% | 1 |
| Croatia | 1,30% | 8 |
| Cyprus | 0,49% | 3 |
| Czechia | 0,81% | 5 |
| Denmark | 0,49% | 3 |
| Egypt | 1,79% | 11 |
| Ethiopia | 0,16% | 1 |
| France | 1,30% | 8 |
| Georgia | 0,65% | 4 |
| Germany | 5,19% | 32 |
| Greece | 3,73% | 23 |
| Hungary | 1,62% | 10 |
| India | 3,57% | 22 |
| Indonesia | 0,32% | 2 |
| Iran | 0,32% | 2 |
| Israel | 0,49% | 3 |
| Italy | 13,96% | 86 |
| Japan | 0,32% | 2 |
| Kazakhstan | 0,32% | 2 |
| Kosovo | 0,16% | 1 |
| Kuwait | 0,16% | 1 |
| Kyrgyzstan | 0,65% | 4 |
| Latvia | 0,49% | 3 |
| Lebanon | 0,16% | 1 |
| Lithuania | 0,32% | 2 |
| Luxembourg | 0,16% | 1 |
| Malaysia | 0,65% | 4 |
| Mexico | 0,97% | 6 |
| Moldova | 1,46% | 9 |
| Montenegro | 0,16% | 1 |
| Morocco | 0,16% | 1 |
| Myanmar (formerly Burma) | 0,32% | 2 |
| Netherlands | 0,49% | 3 |
| Nicaragua | 0,16% | 1 |
| Nigeria | 0,16% | 1 |
| North Macedonia (formerly Macedonia) | 0,81% | 5 |
| Norway | 0,65% | 4 |
| Peru | 0,49% | 3 |
| Philippines | 1,14% | 7 |
| Poland | 2,27% | 14 |
| Portugal | 2,44% | 15 |
| Romania | 3,90% | 24 |
| Russia | 2,76% | 17 |
| Serbia | 1,14% | 7 |
| Slovakia | 0,49% | 3 |
| Slovenia | 0,32% | 2 |
| South Africa | 0,16% | 1 |
| South Korea | 0,81% | 5 |
| Spain | 4,38% | 27 |
| Sudan | 0,49% | 3 |
| Sweden | 0,16% | 1 |
| Switzerland | 1,30% | 8 |
| Taiwan | 0,16% | 1 |
| Tajikistan | 0,16% | 1 |
| Thailand | 0,32% | 2 |
| Tunisia | 0,97% | 6 |
| Turkey | 5,52% | 34 |
| Uganda | 0,16% | 1 |
| Ukraine | 3,57% | 22 |
| United Kingdom (UK) | 2,27% | 14 |
| United States of America (USA) | 1,14% | 7 |
| Uzbekistan | 0,81% | 5 |
| Vietnam | 0,16% | 1 |
| Yemen | 0,16% | 1 |

**Supplemental table 2: The Weighted Average Score (WAS), standard deviation, standard error (SE), and 95% confidence intervals (CI) of SE for vaccine heistancy.**

|  | Average | Variance | Standard deviation | Standard Error | 95% confidence interval | |
| --- | --- | --- | --- | --- | --- | --- |
| **^a^Hesitancy** |  |  |  |  | lower | upper |
| Stroke/vascular neurology | 3.083 | 1.346 | 1.160 | 0.048 | 2.988 | 3.177 |
| Peripheral neuropathy | 2.945 | 1.198 | 1.094 | 0.045 | 2.856 | 3.034 |
| Neuromuscular disoders | 3.131 | 1.283 | 1.133 | 0.047 | 3.039 | 3.223 |
| Movement disorders | 2.677 | 1.079 | 1.039 | 0.043 | 2.592 | 2.762 |
| Multiple sclerosis/Neuroimmunology | 3.719 | 1.195 | 1.093 | 0.045 | 3.631 | 3.807 |
| Dementia and cognitive disorders | 2.874 | 1.231 | 1.109 | 0.046 | 2.784 | 2.964 |
| Epilepsy | 2.920 | 1.217 | 1.103 | 0.046 | 2.830 | 3.010 |
| Headache and pain | 2.632 | 1.191 | 1.091 | 0.045 | 2.544 | 2.721 |
| Neuroinfection | 3.113 | 1.350 | 1.162 | 0.050 | 3.016 | 3.210 |
| Sleep disorders | 2.424 | 0.907 | 0.953 | 0.041 | 2.345 | 2.504 |
| Autonomic nervous system disorders | 2.690 | 1.144 | 1.070 | 0.046 | 2.601 | 2.780 |
| **Distrust in SARS-CoV-2 vaccination** |  |  |  |  |  |  |
| Stroke/vascular neurology | 1.813 | 1.008 | 1.004 | 0.042 | 1.732 | 1.895 |
| Peripheral neuropathy | 1.699 | 0.795 | 0.892 | 0.037 | 1.626 | 1.772 |
| Neuromuscular disorders | 1.795 | 0.935 | 0.967 | 0.041 | 1.716 | 1.875 |
| Movement disorders | 1.704 | 0.883 | 0.940 | 0.040 | 1.626 | 1.782 |
| Multiple sclerosis/neuroimmunology | 2.092 | 1.186 | 1.089 | 0.045 | 2.005 | 2.180 |
| Dementia and cognitive disorders | 1.827 | 0.949 | 0.974 | 0.041 | 1.746 | 1.908 |
| Epilepsy | 1.789 | 0.925 | 0.962 | 0.041 | 1.709 | 1.869 |
| Headache and pain | 1.724 | 0.892 | 0.944 | 0.040 | 1.646 | 1.802 |
| Neuroinfection | 1.839 | 0.974 | 0.987 | 0.042 | 1.758 | 1.921 |
| Sleep disorders | 1.578 | 0.772 | 0.879 | 0.038 | 1.504 | 1.652 |
| Autonomic nervous system disorders | 1.684 | 0.902 | 0.950 | 0.041 | 1.605 | 1.764 |
| **Fear of adverse events of vaccination.** |  |  |  |  |  |  |
| Stroke/vascular neurology | 2.412 | 1.155 | 1.075 | 0.044 | 2.325 | 2.499 |
| Peripheral neuropathy | 2.194 | 1.021 | 1.010 | 0.042 | 2.112 | 2.277 |
| Neuromuscular disorders | 2.322 | 1.079 | 1.039 | 0.044 | 2.236 | 2.407 |
| Movement disorders | 1.949 | 0.994 | 0.997 | 0.042 | 1.867 | 2.031 |
| Multiple sclerosis/neuroimmunology | 2.807 | 1.031 | 1.015 | 0.041 | 2.726 | 2.888 |
| Dementia and cognitive disorders | 1.948 | 1.021 | 1.010 | 0.043 | 1.865 | 2.032 |
| Epilepsy | 2.099 | 0.973 | 0.986 | 0.041 | 2.019 | 2.180 |
| Headache and pain | 1.991 | 0.991 | 0.996 | 0.042 | 1.909 | 2.073 |
| Neuroinfection | 2.277 | 1.117 | 1.057 | 0.045 | 2.190 | 2.364 |
| Sleep disorders | 1.761 | 0.941 | 0.970 | 0.042 | 1.680 | 1.843 |
| Autonomic nervous system disorders | 1.899 | 1.024 | 1.012 | 0.043 | 1.814 | 1.983 |
| **Fear of disease reactivation/ worsening** |  |  |  |  |  |  |
| Stroke/vascular neurology | 2.121 | 1.286 | 1.134 | 0.048 | 2.028 | 2.215 |
| Peripheral neuropathy | 2.224 | 1.156 | 1.075 | 0.045 | 2.136 | 2.313 |
| Neuromuscular disorders | 2.343 | 1.231 | 1.109 | 0.047 | 2.252 | 2.435 |
| Movement disorders | 1.841 | 0.969 | 0.984 | 0.042 | 1.758 | 1.923 |
| Multiple sclerosis/neuroimmunology | 2.982 | 1.085 | 1.041 | 0.043 | 2.898 | 3.065 |
| Dementia and cognitive disorders | 1.756 | 0.930 | 0.964 | 0.041 | 1.675 | 1.838 |
| Epilepsy | 2.108 | 1.081 | 1.040 | 0.044 | 2.022 | 2.194 |
| Headache and pain | 1.953 | 1.063 | 1.031 | 0.044 | 1.867 | 2.039 |
| Neuroinfection | 2.273 | 1.205 | 1.098 | 0.047 | 2.181 | 2.364 |
| Sleep disorders | 1.628 | 0.814 | 0.902 | 0.039 | 1.551 | 1.705 |
| Autonomic nervous system disorders | 1.878 | 0.985 | 0.993 | 0.043 | 1.795 | 1.962 |

^a^In your experience, people with the following neurological conditions are more hesitant to receive SARS-CoV-2 vaccination than healthy people of the same age.
