# Supplementary material for: Is there still hesitancy towards SARS-CoV-2 vaccination among people with neurological disease– a survey of the NeuroCOVID-19 task force of the European Academy of Neurology
Source: Neurol Sci. 2025 Feb 4;46(4):1467–76. doi: 10.1007/s10072-025-08017-w (PMC11920348; doi:10.1007/s10072-025-08017-w)
Supplement: Supplementary file 2 — Supplementary Material 2 Questionaire [file 10072_2025_8017_MOESM2_ESM.docx]

The following neurological conditions currently lead to restricted access to SARS-CoV-2 vaccination. (0 = I do not know; 1 = I strongly disagree; 2 = I disagree; 3 = Neither agree nor disagree; 4 = Agree; 5 = Strongly agree)

In your experience, people with the following neurological conditions are more hesitant to receive SARS-CoV-2 vaccination than healthy people of the same age. (0 = I do not know; 1 = I strongly disagree; 2 = I disagree; 3 = Neither agree nor disagree; 4 = Agree; 5 = Strongly agree)

Please provide a score on the reasons for vaccine hesitancy among people with the following neurological conditions. (0 = Not important at all; 1 = Of little importance; 2 = Important; 3 = Very important; 4)
